# Angiotensin-Converting Enzyme (ACE)-Inhibitor Activity of Novel Peptides Derived from Porcine Liver and Placenta

**DOI:** 10.3390/molecules30030754

**Published:** 2025-02-06

**Authors:** Nicholas A. Pearman, Gordon A. Morris, Alan M. Smith

**Affiliations:** 1 Department of Physical and Life Sciences, School of Applied Sciences, University of Huddersfield, Huddersfield HD1 3DH, UK; 2 Department of Pharmacy, School of Applied Sciences, University of Huddersfield, Huddersfield HD1 3DH, UK

**Keywords:** novel peptides, meat waste, hydrolysates, in silico digests, ACE inhibitor

## Abstract

Peptides isolated from various biological materials are potential sources for novel angiotensin-converting enzyme (ACE) inhibitors. Here, the ACE-inhibitory activity of peptides derived from papain-digested hydrolysates of porcine liver and placenta were investigated. A high-throughput method was developed to identify potential bioactive peptides from the hydrolysates using in silico enzymatic cleavage, HPLC-MS/MS, and bioinformatics tools. Four peptides (FWG, MFLG, SDPPLVFVG, and FFNDA) were selected based on their predicted bioactivity, then synthesised and tested for ACE inhibition. All samples demonstrated ACE-inhibitory activity, with FWG and MFLG showing greater potency than SDPPLVFVG and FFNDA. The placenta hydrolysate outperformed both the liver hydrolysate and synthetic peptides in ACE inhibition, possibly due to it containing a higher proportion of dipeptides. The synthetic peptides’ IC50 values were comparable to those reported for porcine muscle-derived peptides in previous studies. While less potent than the commercial ACE inhibitor captopril, the identified peptides showed promising ACE-inhibitory activity. This research demonstrates the potential of porcine liver and placenta as sources of novel ACE-inhibitory peptides and highlights the effectiveness of the developed high-throughput method for identifying bioactive peptides; this method could subsequently be adapted to other peptide sources, facilitating the development of innovative functional foods or nutraceuticals.

## 1. Introduction

Bioactive peptides (BAPs) can be released from precursor proteins by proteolytic enzymes. Proteases used in the hydrolysis of these precursor proteins include the digestive enzymes trypsin and pepsin. Other such proteases include papain (from papaya), and bromelain (from pineapple), which are used in meat tenderisation and are derived from plants [1,2]. Hydrolysates obtained from these protein digests can be fractionated using techniques such as fast protein liquid chromatography (FPLC) or reverse-phase high-performance liquid chromatography (RP-HPLC). The fractions obtained can then be assayed for bioactive properties such as anti-angiotensin-converting enzyme (ACE) activity. Any active fractions can be further purified using RP-HPLC to isolate individual peptides. These peptides can then be sequenced de novo using MS/MS [3,4].

Peptides identified by de novo sequencing can then be cross-checked against repositories/databases of sequences of known bioactive peptides [5]. The desired outcome is to discover novel bioactive peptides not found in these repositories/databases and subsequently determine their bioactivities. This de novo sequencing approach can also be used to identify the originating protein from which specific peptides were derived. Identification of the originating protein in crude hydrolysates is useful in understanding their composition. Despite knowing the source of the hydrolysate raw material, quantifying the proportion in which the original protein that released a specific BAP was present in the bulk hydrolysate can be difficult. However, knowing the source proteins makes it possible to perform in silico digests, using online tools such as BIOPEP [6] to generate a list of peptides that could be released following different enzymatic digestions with various proteases. Moreover, as protease enzymes cleave at different positions in the amino acid chains, multiple peptide fragments can be generated, and these will consequently have different levels and modes of bioactivity [7].

This method can be used to identify interesting peptides that not only have a high chance of demonstrating bioactivity, but also are novel. The theoretical peptides generated using this method can then be synthesized and experimentally analysed for potential bioactivity. Recently, we have investigated BAPs derived from the papain hydrolysis of porcine liver. Novel bioactive peptides were isolated; the source protein was identified using the BIOPEP database [6]; and bioactivity potential was determined using the Peptide Ranker tool [5]. The peptides identified were synthesised and then shown to have antioxidant activity comparable to that of Trolox [8]. Here, we explore the potential of these novel peptides and the crude hydrolysates from which they were derived as angiotensin-converting enzyme (ACE) inhibitors. Several studies have been performed on the ability of specific peptides and protein hydrolysates to inhibit ACE in vitro. Indeed, the basic structure of ACE inhibitors and their mechanisms of action are well known [9,10]. Knowledge of these structures can be used to select previously un-reported/novel peptides that score highly on the Peptide Ranker tool and that have chemical attributes similar to those of known ACE inhibitors.

The aim of this research was to evaluate hydrolysates derived from meat waste material (liver and placenta) for bioactivity, specifically, ACE inhibition. This was divided into two areas of investigation: the bioactivity demonstrated by the crude hydrolysates and the analysis of the structures of these hydrolysates, with the second area leading to the generation of novel synthetic peptides that could also be tested for bioactivity.

## 2. Results

### 2.1. ACE-Inhibition Activity of Crude Liver and Placenta Hydrolysates

**Figure 1:**
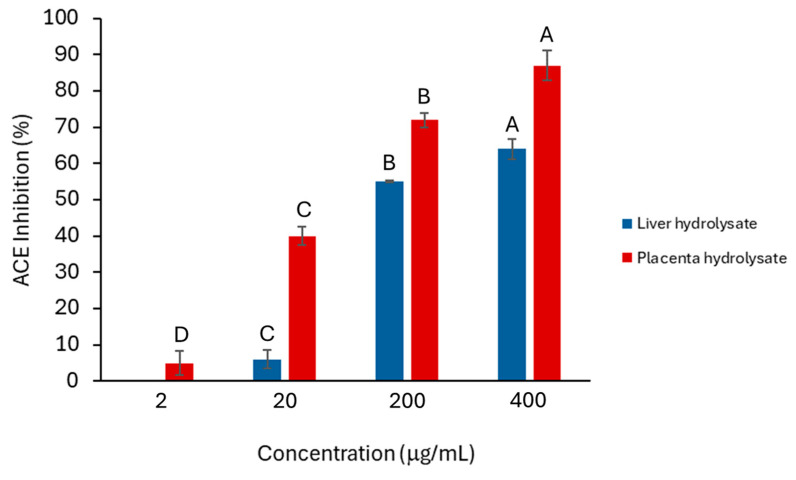
Percentage inhibition of ACE by placenta (red) and liver hydrolysates (blue). Error bars represent one standard deviation. For each hydrolysate, different letters (A, B, C and D) indicate significant differences at 95% confidence (*n* = 2).

**Figure 2:**
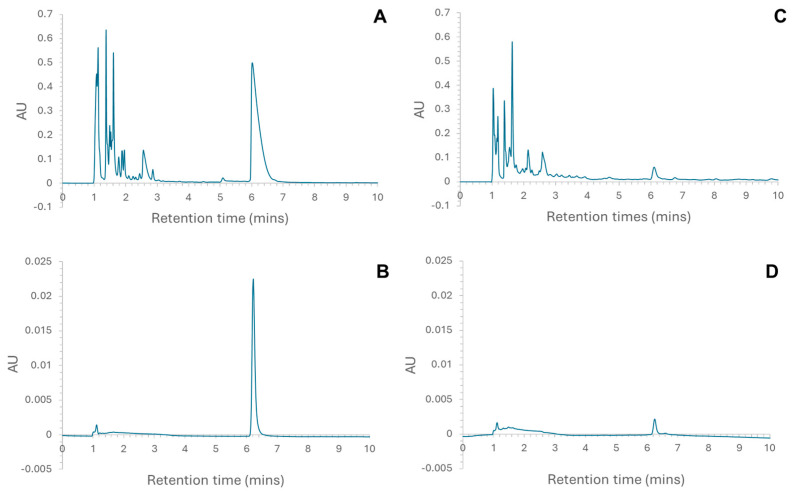
Chromatograms indicating the fraction that was collected for (**A**) liver hydrolysate (peak at 6.017 min); (**B**) the collected fraction re-loaded onto the column to check for purity (peak at 6.217 min); (**C**) placenta hydrolysate (peak at 6.100 min); and (**D**) the collected fraction re-loaded onto the column to check for purity (peak at 6.250 min).

The placenta hydrolysate showed significantly more anti-ACE activity than did the liver hydrolysates (Figure 1), and the activity of both hydrolysates was concentration dependent. A common feature of peptides that show anti-ACE activity is that they are often dipeptides. Moreover, HPLC analysis also indicated that there was a larger proportion of smaller peptides in the placenta hydrolysate than in the liver hydrolysates (Figure 2A,C). This would explain why the placenta hydrolysate showed significantly (*p* < 0.05) higher activity than the liver samples at all concentrations.

### 2.2. Peptide Sequencing Using MS/MS

RP-HPLC was used to fractionate the liver and placenta hydrolysates, and the eluent corresponding to a peak at ~6 min (Figure 2) was collected. These were expected to contain larger peptides not previously reported; furthermore, for the liver hydrolysate, this is the most prominent peak. The initial analytical HPLC analysis of the liver (Figure 2A) and placenta (Figure 2C) confirmed that the hydrolysates were made up of a complex mixture of peptides. The majority of peptides eluted in the first 4 min. This shows that the majority of the peptides, for both liver and placenta hydrolysates, were small, though a greater proportion of the placenta hydrolysate was eluted prior to 4 min. Peptide chain length is only one of several factors affecting retention time of peptides in RP-HPLC; others include polarity, position of individual amino acid residues, and total charge. It is suggested that the lower molecular weight of digested peptides not only ensures that bioactive sequences are exposed, but renders them also more biologically available because they can easily be absorbed into the body [11]. Samples from the collected peaks were analysed on the RP-HPLC using the same parameters used to check the purity of the aliquots (Figure 2B,D), and the chromatograms indicated that the aliquots were pure. In both chromatograms (Figure 2B,D), the small peak at 1.117 min was attributed to the solvent peak. These pure fractions were then further analysed using MS/MS, and the following peptides were identified in the samples [8]:

Liver peak at ~6 min (L6): TPANEMTPTR and SAADKANVKAA

Placenta peak ~6 min (P6): YSGTGQQQPER

The peptides were cross-referenced with known peptide sequences using the UniProt database [12] to determine their proteins of origin. The originating proteins were identified as cytosol aminopeptidase, haemoglobin subunit alpha, and Type VI collagen alpha-1 chain for TPANEMTPTR, SAADKANVKAA, and YSGTGQQQPER, respectively [8].

### 2.3. In Silico Papain Digestion and Activity Prediction

Peptides generated from the simulated papain digests of cytosol aminopeptidase, haemoglobin subunit alpha (liver hydrolysate source) and Type VI collagen (partial) alpha-1 chain (placenta hydrolysate source) were ranked for potential activity using the Peptide Ranker tool by Bioware [5]. Most of the fragments from all the simulated digests were single amino acids and dipeptides () that were excluded from selection due to previously reported bioactivity. The most potent peptide predicted that had not been previously described in the literature was FWG from cytosol aminopeptidase, with a score of 1.00; it was followed by MFLG from haemoglobin subunit alpha, with a score of 0.98, FFNDA from Type IV collagen partial alpha-1 chain (0.77), and SDPPLVFVG from cytosol aminopeptidase, with a score of 0.75. Predicted peptides with a predicted rank ≥ 0.65 for all the digests are displayed in Table 1.

### 2.4. Synthesised Peptides

Using the potential activity predicted by the Peptide Ranker tool, the peptides indicated in Table 1 were selected to be synthesised and analysed (the originating protein, hydrolysate source, and Peptide Ranker score are also reported): FFNDA type VI collagen alpha-1 chain from placenta hydrolysate (0.77), MFLG haemoglobin subunit alpha from liver hydrolysate (0.96), SDPPLVFVG cytosol aminopeptidase from liver hydrolysate (0.75), and FWG cytosol aminopeptidase from liver hydrolysate (1.00). These peptides were chosen because they all scored relatively high in the Peptide Ranker tool and have not been previously reported in the literature as having ACE-inhibitor activity.

### 2.5. ACE Inhibition Assay of Synthetic Peptides

**Figure 3:**
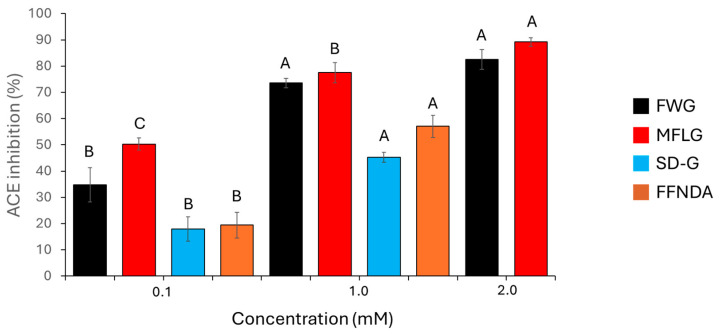
Percentage inhibition of ACE by the synthetic peptides FWG, MFLG, SDPPLVFVG (SD-G), and FFNDA. Error bars represent one standard deviation. For each synthetic peptide, different letters (A, B and C) indicate significant differences at 95% confidence (*n* = 3).

The synthetic peptides had some inhibitory activity against ACE. This activity was concentration-dependent, and Figure 3 shows the percentage ACE inhibition of these synthetic peptides. MFLG and FWG showed a greater inhibitory effect than did SDPPLVFVG or FFNDA. SDPPLVFVG and FFNDA were not assayed at 2 mM, as there was not enough peptide to prepare this concentration. All synthetic peptides tested showed considerably less ACE-inhibition activity than captopril; 0.1 μM and 0.01 μM captopril had ACE-inhibition activities of 98.7% and 88.7%, respectively.

To compare the synthetic peptides used in this study with peptides used in similar investigations, the inhibitory concentration at 50% (IC50) values were calculated (Table 2). It is useful to express these data as IC50, as it can be difficult to determine the point at which a substance is 100% effective.

## 3. Discussion

The aim of this study was to develop a simple protocol to identify bioactive peptides in meat-waste hydrolysates, one example being peptides showing ACE-inhibition activity. Previous researchers have used a similar premise to identify bioactive peptides. However, in the main, they started with peptides that had been isolated from active fractions [26,27,28]. This new approach was designed to allow for higher throughput. Through sequencing the peptides directly after an initial separation, then using the BIOPEP and Peptide Ranker tools, it was possible to identify potentially bioactive peptides prior to testing bioactivity. Although with the caveat that, it has previously been demonstrated that there are significant differences between predicted bioactive peptides, and peptides released after hydrolysis [29]. This is in part due to the amount of information available in bioactive peptide databases; however, this was not an issue in this study, as the objective was to identify novel bioactive peptides, which would not appear in these databases. Clearly, taking this into account, it is possible (or even likely) that peptides released from an in vitro enzyme digestion could/would be different from those predicted using an in silico approach. This could be the result of, for example, a miscleavage due to variations in the quality of the enzymes used [30], although the secondary, tertiary or quaternary structures of the proteins under investigation is also important, as these can prevent the enzyme from accessing some peptide bonds. It is expected that the cleavages associated with the experimental process will be non-specific, and the complexity of the peptide profile cannot be attributed solely to the enzyme (i.e., papain) [31].

The host protein was used as a starting point for in silico enzymatic cleavage in the present study, as the probability of identifying novel bioactive peptides from the HPLC MS/MS process is small. Indeed, it may not be an efficient method by which to directly identify bioactive peptides. None of the peptides identified from the MS/MS data (TPANEMTPTR, SAADKANVKAA, and YSGTGQQQPER) showed any similarities with those previously reported as being bioactive when they were analysed using the BIOPEP database. Furthermore, the Peptide Ranker predicted that all the peptide sequences had a low probability of being bioactive. While it is not unexpected that the peptides identified using MS/MS would show no potential bioactivity, knowledge of the originating proteins could then be used for in silico analysis in the search for potentially novel bioactive peptides. Once the originating protein has been identified, it may be expected that a specific bioactive peptide will be present in the hydrolysate. An example of this is the prevalence of ACE inhibitors identified in hydrolysates from a range of animal sources.

An active peptide (LLMLDNDLPP) was isolated from Pacific cod-skin gelatine after hydrolyses (pepsin, trypsin, and alpha chymotrypsin), and it showed potent non-competitive ACE inhibition, in addition to antioxidant properties [26]. Indeed, peptides generated using gastrointestinal enzymes have been shown to contain hydrophobic residues at specific positions in their sequence. Structural features that enable potential ACE-inhibition activity include branched-chain aliphatic amino acids at the N-terminus (leucine and methionine), and proline and leucine residues at the C-terminus. Tyrosine, phenylalanine, tryptophan, isoleucine, valine, and arginine have also been reported to have an important effect on ACE binding [32]. It has also been suggested that the low molecular weight of digested peptides not only allows the bioactive sequences to be exposed, but also renders them more bioavailable because they can be more readily absorbed in vivo [33]. Moreover, the peptide (VLAQYK) derived from meat waste had the greatest ACE-inhibitory activity. This was reportedly due to the presence of valine at the N-terminus [7]. The fact that bioactive peptides tend to terminate in hydrophobic residues is a significant factor in their activity. The aromatic side chain on tyrosine may also cause steric hindrance, whilst the smaller valine residue binds to the ACE. In the investigation of hydrolysates from thornback ray skin, the most active ACE inhibitor was found to be GIPGAP [26]. Other peptides from this hydrolysate were LLMLDNDLPP and VLAQYK, both of which have hydrophobic sidechains at the N-terminus (LLML, and VLA), whilst LLMLDNDLPP and GIPGAP both have proline at the C-terminus. Indeed, proline has been shown to be an important amino acid in the activity of captopril and is considered to be of importance in the inhibition of ACE [34].

All samples tested in the present study showed at least some anti-ACE activity. There were clear differences between individual synthetic peptides. FWG and MFLG showed greater activity than did SDPPLVFVG or FFNDA. At the lowest concertation tested (0.1 mM), MFLG showed significantly more activity than FWG; however, this was not observed at higher concentrations (1 mM and 2 mM). Evidence suggests that FWG and MFLG were readily soluble in aqueous solution, whereas SDPPLVFVG and FFNDA were perhaps less soluble. This could, in part, explain their lower anti-ACE activity, although at the concentrations studied (0.1 and 1.0 mM), this would not be expected to be significant. Interestingly, all the synthetic peptides contained phenylalanine, which has previously been linked to strong ACE-inhibitory activity [35].

Comparing the synthetic peptides (Figure 3) with the activity of the hydrolysates from which they were derived (Figure 1), it was evident that the placenta hydrolysate outperformed both the liver hydrolysate and the synthetic peptides in inhibiting ACE (in the case of all four synthetic peptides, a concentration of 1 mM is greater than 400 mg/mL). Since the placenta hydrolysate contained a larger proportion of dipeptides, it would be expected to have a greater effect on ACE inhibition. However, both the liver and placenta hydrolysates had a greater ACE-inhibitory effect at higher concentrations when compared to snail protein hydrolysates of a similar concentration [36]. Further studies should incorporate bioavailability and/or in vivo studies to further support this data.

Selected data, examined with the aim of investigating ACE-inhibitory peptides from porcine proteins [13,14,15,16,17,18,19,20,21,22,23,24,25], indicated that peptides obtained from porcine sources had an ACE-inhibitory IC50 ranging from 1 to 1194 µM (Table 2). These values are consistent with those of the peptides analysed here, where MFLG (70 µM) performed best among the four synthetic peptides.

Captopril concentrations of 0.1 µM and 0.01 µM reduced ACE activity by 98.7% and 88.7%, respectively (), making captopril more potent than any of the synthetic peptides investigated in this study; these values are consistent with the IC50 values measured previously [25]. The observation that synthetic peptides were less potent than captopril is not surprising when the structures of these synthetic peptides are compared with those of specifically designed ACE inhibitors. The synthetic peptides used in this research were selected not because they are potent anti-ACE inhibitors, but due to their potential but non-specified bioactivity. While they do show some anti–ACE activity, their activities are low in comparison with those of commercially available ACE inhibitors. Therefore, the bioactivity predicted by the Peptide Ranker tool is unlikely to be associated with ACE inhibition. The tools used to select peptides for synthesis could be used more specifically to focus on specific peptide sequences that have been reported to have anti-ACE activity. It is important to note, however, that the BIOPEP tool used in this study is regularly updated with information about new sites susceptible to proteolysis in protein sequences [37]. Therefore, updated versions of the BIOPEP tool may result in predicted hydrolysis of the target proteins that is different from the patterns generated by earlier versions of the software.

## 4. Materials and Methods

Liver and placenta hydrolysates were produced by and supplied by Biofac A/S (Kastrup, Denmark). They were produced by processing raw materials (liver or placenta) before hydrolysis was carried out with the cysteine protease papain. Briefly, the raw material generated from meat waste was homogenised before the addition of water and heating to 85 °C. Hydrogen peroxide was then added to the mix, and the batch was heated to 63–68 °C. At this point, papain was added and the reaction left for 10–14 h. The temperature then increased to 85 °C for 30 min to inactivate the enzyme, after which several filtration steps were undertaken to purify the sample. The sample was then dried through evaporation and spray-dried before the final sieving process. Preliminary characterisation of the liver and placenta hydrolysates showed that they contained very little carbohydrate material (2.5 ± 0.6 and 0.6 ± 0.5%) and had weight-average molar masses of 3.5 ± 1.4 and 5.8 ± 2.1 kg/mol, respectively. Data from the stability studies indicate that hydrolysates are stable in solution (pH 7.4) over the period (168 h) that the study was performed. Synthetic peptides (FWG, MFLG, FFNDA, and SDPPLVFVG) were supplied (Protein Peptide Research Ltd., Fareham, UK). All other general laboratory reagents and materials used were supplied by Sigma Aldrich (Gillingham, UK), Sarsedt (Nümbrecht, Germany) and Thermo Scientific (Loughborough, UK).

### 4.1. Isolation of Individual Fractions for MS Analysis

Liver hydrolysate and placenta hydrolysate solutions (5 mg/mL) were prepared in aqueous HPLC running buffer (ultrapure water, acetonitrile (0.2%), TFA (0.01%)) and filtered using 0.2 µm syringe filters. These were loaded onto an analytical HPLC column (Ascentis^®^ Express Peptide ES-C18) using a 20 µL loading loop at a flow rate of 1 mL/min. The concentration gradient of acetonitrile for all samples increased from 5% to 29% over 10 min and then increased from 29% to 95% over 5 min. Samples were collected manually and verified by running the collected sample through the same HPLC setup to ensure that it contained only material from the specific peak. Sample L6a was derived from the liver hydrolysate (L) and was collected from the fraction eluted at 6.017 min. Sample L6b was collected from a similar fraction, from the same hydrolysate (L), but on a different run. This was done to evaluate the reproducibility of the method. Sample P6 was derived from the placenta hydrolysate (P) and was collected from the eluent corresponding to the peak at 6.1 min. The concentrations of the peptides in the samples isolated from the peak at 6.1 min were estimated using a standard curve. Peptide solutions (80, 40, 20, and 10 µg/mL) were prepared using the aqueous buffer with 5% acetonitrile, and the samples were analysed using a UV-Vis spectrophotometer at 220 nm.

### 4.2. Peptide Sequencing Using MS/MS

The three samples that were collected (L6a, L6b, and P6) were shipped on dry ice to the Metabolomics & Proteomics Lab at the University of York for peptide sequencing using LC-MS/MS using a Bruker maXisHD mass spectrometer interfaced to a 50 cm PepMap column with a Bruker CaptiveSpray ion source. Peptides were eluted from the column over a 35 min gradient at 300 nL/min. Eluting peptides were selected for MS2 fragmentation using top-speed data-dependent acquisition, a dynamic 7–25 Hz acquisition rate, and 1 s cycle time. Product ion spectra were searched against the porcine subset of the UniProt database [10] using Mascot, with no enzyme specificity. Search results were filtered to require expect scores of 0.05 or lower, such that sequence matches had a chance equal to or less than 1 in 20 of being a false positive, and therefore incorrect.

### 4.3. In Silico Digestion, and Activity Prediction

In silico enzyme digestion were carried out on the protein sequences using the analysis tool available on the BIOPEP website [6]. Sequences were generated using papain and then cross-referenced against a number of bioactive-peptide databases to find any active sequences that had been previously reported; these were then excluded. Peptide sequences that had not been reported were then analysed using the Peptide Ranker tool by Bioware [5]. Peptide Ranker predicts the likelihood of a peptide sequence being bioactive (0.00 being highly unlikely, 1.00 being highly likely).

### 4.4. Peptide Synthesis and Preparation

Based on the in silico activity prediction, four synthetic peptides were ordered from Peptide Synthetics (Protein Peptide Research Ltd., Fareham,, UK). They were supplied as 10 mg aliquots of lyophilised powders. The synthetic peptides were dissolved in deionised water to produce 10 mM stock solutions:

- FWG: 10 mg/mL = 24.48 mM therefore 10 mM = 4.085 mg/mL
- MFLG: 10 mg/mL = 21.43 mM therefore10 mM = 4.666 mg/mL
- SDPPLVFVG: 10 mg/mL = 10.75 mM therefore 10 mM = 9.302 mg/mL
- FFNDA: 10 mg/mL = 16.32 mM or10 mM = 6.127 mg/mL

The peptide solutions were aliquoted and stored at −20 °C prior to use.

### 4.5. Angiotensin-Converting Enzyme (ACE)-Inhibition Assay

The ACE-inhibitory activity was determined using a modified Cheung and Cushman (1971) method, which utilises the ACE-specific substrate hippuryl histidyl leucine (HHL) [38]. ACE from rabbit lung (purchased from Sigma Aldrich 2U equal to 1 mg of protein) was reconstituted in milli-q water (1 mL) to produce the ACE stock solution (1 U/mL, 500 μg/mL of protein). ACE working solution (200 mU/mL, 100 μg/mL of protein) was prepared by diluting the stock solution 1:5. ACE stock solution was stored at −20 °C, and the ACE working solution was prepared when required. HHL solution was prepared by adding HHL (2.15 mg) to TRIS buffer (pH 8.3) (10 mL) containing NaCl (300 mM), and ZnSO4 (10 μM) (10 mL). Peptide hydrolysate preparations were prepared by dissolving crude liver hydrolysate into milli-q water. Milli-q water or experimental sample (40 μL) and 0.5 mM HHL (145 μL) were added to 1.5 mL micro-centrifuge tubes and incubated in a water bath at 37 °C for 3 min; then, 200 mU/mL ACE (15 μL) was added to each tube and the tubes were then incubated in a water bath at 37 °C for 30 min. After 30 min, 1 M HCl (200 μL) was added to the tubes to stop the reaction. Experimental sample preparation is illustrated in Table 3, where column A shows experimental samples, column B shows control 1, and column C shows control 2. Captopril was used as a known ACE inhibitor.

To determine the anti-ACE activity of the analytes, the samples were analysed using HPLC (Beckman Coulter) fitted with an Ascentis^®^ Express Peptide ES-C18, 2.7 μm column. The samples were eluted with an isocratic mobile phase consisting of 50% methanol and 50% water containing trifluoroacetic acid (TFA) (0.2%) at a flow rate of 0.4 mL/min. The substrate and the product were detected using UV at 228 nm. The ability of each peptide to inhibit ACE was measured by comparing the reduction of HA (the product of ACE digested HHL) in relation to the positive control (no inhibitor or peptide present). Inhibition was calculated using Equation (1), as follows:

ACE inhibition (%) = (1 − (B ÷ A)) × 100(1)

where A is the area of the HA peak in control 1 and B is the area of the HA peak in experimental sample. To assess the anti-ACE activity of the synthetic peptides, samples were prepared by diluting the 10 mM stock synthetic peptide solutions using milli-q water to produce samples at 2 mM, 1 mM, and 0.1 mM. SDPPLVFVG and FFNDA were not assayed at 2 mM because there was not enough material to generate data at that concentration.

### 4.6. Statistical Analysis

Statistical analyses were performed in duplicate or triplicate using Minitab (version 19) with a *p* value < 0.05 considered significant. Standard deviations derived from the mean values are indicated with error bars. One-way analysis of variance (ANOVA) showing grouping information using a post-hoc Tukey method and 95% confidence level was used to show significant differences between groups.

## 5. Conclusions

It was evident in this study that the synthetic peptides and precursor hydrolysates did show some ACE activity, with the MFLG and placenta hydrolysate demonstrating the greatest effect, although both were much less potent than captopril. It should be noted, however, that the synthetic peptides and precursor hydrolysates were not selected specifically for anti-ACE activity, so the relatively good performance of MFLG and placenta hydrolysate is encouraging. Moreover, these data suggest it is likely that there are potent anti-ACE peptides in the hydrolysate. What is clear is that there is potential to use this method to identify novel peptides with potent bioactivity from in silico digests of known proteins (using specific enzymes) that can be subsequently synthesised and experimentally analysed for activity. The results of this study with regard to the characterisation and identification of the peptides in meat protein hydrolysates indicate that there is potential to further refine the hydrolysates to isolate more active components. This information could be used to either modify/fine-tune the manufacturing process to create a product with added value or could be used by primary customers for their products. The process used to predict bioactive peptides present in these hydrolysates is a useful development in the discovery of novel bioactive peptides. This method could identify potentially interesting peptide targets prior to the use of the time-consuming processes involved in traditional empirical analysis. The limited scope of this study led to only a small number of peptides being investigated. However, it is probable that there are several more novel bioactive peptides present in both the liver and placenta hydrolysates. From a commercial point of view, this is interesting, as currently available synthetic drugs, e.g., captopril, can cause allergic reactions and hence there is a demand for ACE-inhibitory peptides derived from natural sources [19] and from food-waste materials [39].

## Figures and Tables

**Table 1:** Predicted peptides with a prediction rank ≥ 0.65 for all the digests (proteases and proteins). Asterisks indicate peptides that had not been previously reported as being bioactive and that were therefore selected for synthesis. Dipeptides were excluded from the ranking. Adapted from ref. [8].

	Papain Digestion	
Cytosol Aminopeptidase- Derived Peptides	Haemoglobin Subunit Alpha-Derived Peptides	Type IV Collagen Alpha-1 Chain Partial-Derived Peptides
FWG (1.00) *	MFLG (0.96) *	FFNDA (0.77) *
FLLR (0.86)	FPH (0.94)	FSSPA (0.65)
WQR (0.84)	FLA (0.86)	-
FLK (0.81)	-	-
NWH (0.78)	-	-
SDPPLVFVG (0.75) *	-	-

**Table 2:** IC50 values for the synthetic peptides and other porcine-derived peptides reported in the literature. The commercially available drug captopril has been included for comparison.

Peptide *	ACE Inhibition IC_50_ (μM)	Protein Source	References
FWG	470	Cytosol aminopeptidase	This study
MFLG	70	Haemoglobin subunit alpha	This study
SDPPLVFVG	1160	Cytosol aminopeptidase	This study
FFNDA	830	Type IV collagen alpha-1 chain	This study
ITTNP	549	Myosin	[13,14]
MNPPK	946	Myosin	[13,14]
MNP	67	Myosin	[13,14]
NPP	291	Myosin	[13,14]
PPK	>1000	Myosin	[13,14]
ITT	678	Myosin	[13,14]
TTN	673	Myosin	[13,14]
TNP	207	Myosin	[13,14]
RMLGQTPTK	34	Troponin C	[13,15]
RMLGQTP	503	Troponin C	[13,15]
KRVITY	6	Myosin	[16]
VKAGF	20	Actin	[16]
VAP	1	Tropoelastin	[17]
APG	1194	Tropoelastin	[17]
GAP	35	Tropoelastin	[17]
VSP	10	Tropoelastin	[17]
TRP	1	Tropoelastin	[17]
LSP	2	Tropoelastin	[17,18]
VGP	26	Tropoelastin	[17,18]
VLP	4	Tropoelastin	[17,19]
GVG	369	Tropoelastin	[17]
GLG	485	Tropoelastin	[17]
VKKVLGNP	29	Myosin (light chain)	[20,21]
GF(Hyp)GP	91	Collagen	[22]
KRQKYDI	27	Troponin	[17,20]
RPR	382	Nebulin	[20,23]
KAPVA	47	Titin	[20,24]
PTPVP	256	Titin	[20,24]
Captopril	5 × 10^−4^	Commercial	[25]

* For consistency, dipeptides are not reported.

**Table 3:** Sample preparation for angiotensin II-converting enzyme (ACE)-inhibition assay.

Reagents	Volume (μL)		
	**A**	**B**	**C**
Milli-Q Water	0	40	15
Inhibitor/Peptide	40	0	40
0.5 mM HHL	145	145	145
	Incubate at 37 °C for 3 min		
200 mU/mL ACE	15	15	0
	Incubate at 37 °C for 30 min		
1 M HCl	200	200	200

## Data Availability

The original contributions presented in this study are included in the article. Further inquiries can be directed to the corresponding authors.
